# Survival analysis in clinical practice: analyze your own data using an Excel workbook

**DOI:** 10.3325/cmj.2016.57.77

**Published:** 2016-02

**Authors:** Marko Lucijanić

**Affiliations:** Department of Hematology, University Hospital Dubrava, Zagreb, Croatia *markolucijanic@yahoo.com*

Questions about patient survival or the time to disease progression are of the greatest interest in everyday clinical practice, especially when analyzing information about your own patients. Such data are censored in nature (have uneven follow-up times and a variable number of patients who are deceased, lost, or still in follow-up) and cannot be adequately summarized or compared unless specially designed statistical methods are utilized. Such methods are referred to as survival analysis.

## Survival analysis and log-rank test

Survival analysis presented in this article and its supplementary file (Supplementary material(web extra material 1)) is based on the method by Kaplan and Meier (1). In short, two entries about each patient are required – the duration of patient’s follow-up and the patient’s status regarding the event of interest occurring during the follow-up (binary outcome labeled 1 or 0 depending if the patient is deceased or alive, relapsed or still in remission, etc). During calculations, patients are lined-up depending on the follow-up duration. Every time a patient dies/attains an event of interest, a new time interval is formed (visible later as a drop in the survival curve) and the proportion of patients dying is calculated by dividing the number of deaths (usually 1) with the number of patients at risk (current number of patients in follow-up) at the given time. For every time interval, the **cumulative** proportion dying is also calculated by multiplying the proportion dying by the proportions dying at all previous intervals (because the patient had to live through the risk of dying at all previous time intervals) and this information is plotted on a survival curve in a step-wise fashion. The proportion dying and the proportion surviving add up to 100% and the proportion surviving/survival curve is easily created in a similar way. Toward the end of the survival curve, every death has a more pronounced impact on survival because it is divided by a smaller number of patients at risk. If the last patient in the follow-up dies, the survival curve reaches the bottom (0% surviving at this time point).

However, the main interest is how survival curves differ between two or more groups of patients. Survival curves can be easily compared using the log-rank test (2,3). There are several variations of this test with subtle differences in their calculations, consequently producing slightly different end results, which will be analyzed in detail in another future discussion. In general, the log-rank test uses the total observed and the total expected number of deaths in each group (constructing numbers depending on the number of patients at risk in subsequent time intervals) to derive a test statistic to be later analyzed using the χ^2^-test.

Slopes of survival curves represent hazards or death rates at a given moment of time. It is important to note that the main assumption behind the log-rank test is that hazards are proportional in two groups during time, ie, survival curves do not cross each other. If this is not the case, the log-rank test should not be used to compare two groups over a whole time period. If the assumption of proportional hazards is valid, a special statistic, called the hazard ratio, can be calculated, which describes how many times the risk of dying is increased in one of the groups in comparison to the other.

## Using available software

Data analysis of this kind usually requires commercial statistical packages that might not be readily available, at least not in clinical settings or at a specific computer used for data collection. However, most everyday computers are equipped with word processing and spreadsheet software like Microsoft Office or similar toolsets. With this in mind, the author has prepared a workbook for MS Excel program (ver. 2002, Microsoft, Redmond, WA, USA) as a supplementary file accompanying this article.

This workbook utilizes the most widely used Cox-Mantel version of the log-rank test to compare survival curves. It has been in development for more than three years and has been tested on several dozen data sets using commercial statistical packages in order to validate the results. Besides its practical purpose of data analysis, it provides users with direct insight into the mechanics of statistical tests and provides a unique “real-time” learning experience.

## Data preparation, data entry, survival analysis tips, and hints

Before analysis, your data should be recorded in a spreadsheet with the first row containing the variable names and the subsequent rows containing data entries. Survival data should be presented with one numerical variable representing the survival time and one binary variable (ie, 1 or 0) representing the censoring status (dead = 1, alive or lost to follow up = 0). One or more categorical variables representing the factors of interest that could affect survival are also desirable (ie, “sex” variable with entries like “m” and “f”; “age” variable with entries like 1 for younger than 65 years of age and 2 for older, etc). This workbook compares survival in up to four subgroups inside the variables of interest. In addition, it allows an analysis of data sets with up to 300 data entries. These limitations are deliberately made due to the speed of calculations and increased complexity of calculations occurring with the increased number of comparisons. You are still able to compare the entries with more than four subgroups, but you have to select which subgroups you would like to mutually analyze; with such calculations increased vigilance is necessary as false conclusions are more likely.

**Figure 1:**
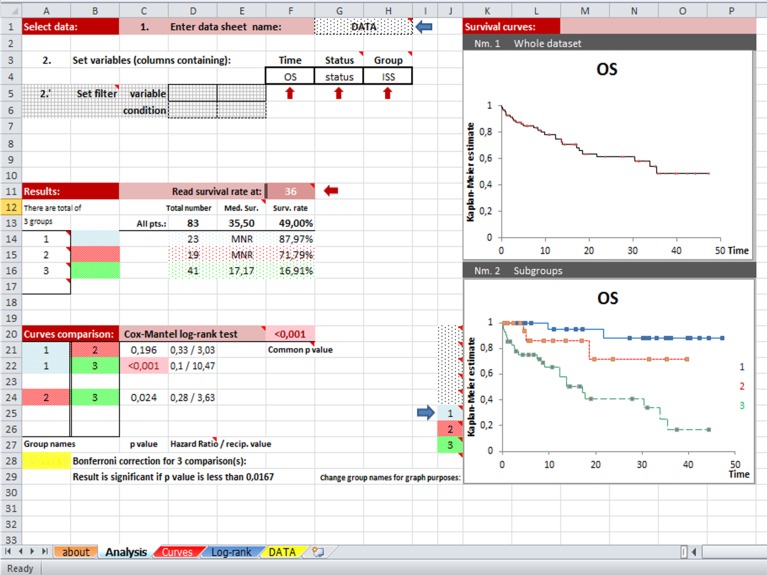
The “Analysis” sheet is divided into four sections: Select data, Results, Curves Comparison, and Survival Curves. Variables are chosen from drop-down menus, which allows for rapid screening through the data containing categorical variables. The significant result is automatically highlighted. Unpublished “real life” data about patients with multiple myeloma are used for presentation.

Data can be simply copy-pasted into the “DATA” sheet, or, in addition, a whole spreadsheet from another workbook can be copied into this one. However, you should select the name of this new spreadsheet in the appropriate cell on the “Analysis” sheet so that the program is able to find your data. Now you can choose the appropriate variables (Time, Status, and Group) from the drop-down menus on the “Analysis” sheet and begin exploring/become acquainted with your data. Two simple data filters have been added, with the ability to select a subset of patients by selecting a preferred variable and a preferred condition (ie, “sex” and “m”). The most useful and most commonly used parameters (number of analyzed patients, median survival time, survival rate at a preferred moment in time, *P* values, and hazard ratios), as well as survival curves for the whole data set and for subgroups are shown on the main analysis sheet (Figure 1). Other parameters (median follow-up time calculated in three different ways, 95% confidence intervals for survival rates, and hazard ratios calculated in two ways – by log-rank and Mantel-Haenszel methods) can be found on the corresponding sheets containing calculations (“Curves” and “Log-rank”). Notice that when a Group variable is chosen, the survival curve for the whole data set is limited only to entries with known data for a chosen parameter. Produced curves are fully customizable, may be enlarged or reduced, and specific details related to analysis may be easily added. Your results can be printed on a single A4 sheet of paper in landscape orientation.

Before leaving the reader to sail on the high seas of statistics, several general principles concerning survival analysis should be emphasized. **First**, survival curves are open-ended if the patient with the longest follow-up is still alive. However, conclusions about surviving after a specific time period should not be drawn from a survival curve if fewer than 5% of all patients are still alive after the time point of interest (to the right of the curve). **Second**, if the assumption of proportional hazards is roughly violated (if survival curves evidently cross during follow-up) the log-rank test and hazard ratios should not be used to describe relations between data (and they will probably yield an insignificant result). In this situation, other statistical tests would be more appropriate. **Third**, when more than two subgroups are present inside Group variable, a common *P* value describing overall effect on survival is provided. Multiple subgroups comparisons are used for *post-hoc* analysis. However, with multiple simultaneous comparisons (three subgroups = three comparisons, four subgroups = six comparisons), the likelihood of detecting falsely significant result increases and correction in the level of the *P* value is needed to accommodate for this phenomenon. This workbook uses the widely accepted Bonferroni correction. Significant results in all described settings will be automatically highlighted in light red for ease of detection.

## Conclusion

This workbook is primarily intended for practicing clinicians who would like to verify their own performance or quickly analyze their own data sets at the moment when the data are collected. It can be used for hypothesis testing or rapid data screening, the choice is yours to make. I hope clinicians will find this tool practical and use it to improve and publish their own results. If you find it useful, please cite this article when reporting your data. However, prior to publishing, your results should be validated using commercial statistical packag
